# Thrombosis in adult primary immune thrombocytopenia in Korea: incidence and risk factors in a 20-year retrospective cohort

**DOI:** 10.1007/s00277-026-07084-z

**Published:** 2026-06-08

**Authors:** Ji Yun Lee, Eun-Jeong Jeong, Woochan Park, Jeongmin Seo, Minsu Kang, Eun Hee Jung, Sang-A Kim, Koung Jin Suh, Ji-Won Kim, Se Hyun Kim, Jeong-Ok Lee, Jin Won Kim, Yu Jung Kim, Keun-Wook Lee, Jee Hyun Kim, Soo-Mee Bang

**Affiliations:** Department of Internal Medicine, Seoul National University College of Medicine, Seoul National University Bundang Hospital, Gumi-ro 173 Beon- gil, Bundang-gu, Seongnam-Si, Gyeonggi-do 13620 Korea

**Keywords:** Immune thrombocytopenia, Thrombosis, Thrombopoietin receptor agonist, Splenectomy, Atrial fibrillation

## Abstract

**Background:**

Immune thrombocytopenia (ITP) is primarily a bleeding disorder, yet thrombotic events occur and remain challenging to predict, particularly in Asian populations and across real-world treatment patterns. We evaluated thrombosis incidence and associated clinical and therapy-related factors in Korean adults with primary ITP.

**Methods:**

We conducted a retrospective cohort study of adults (≥ 19 years) with hematologist-confirmed primary ITP at Seoul National University Bundang Hospital from April 1, 2003 to December 31, 2023. Thrombotic events were identified by diagnostic codes and confirmed by review of imaging and clinical documentation. Patients were classified into five ever-treated groups: never treated (Group 1), first-line only (Group 2), immunosuppressive agents without TPO-RA (Group 3), TPO-RA monotherapy (Group 4), and TPO-RA combination therapy (Group 5). Incidence rates were calculated per 100 person-years (PY). Predictors were assessed using Firth’s penalized logistic regression.

**Results:**

Among 1,038 patients (median age 57 years; 42.6% male), median follow-up was 1.4 years (IQR, 0.3–4.5) with 3,185 PY. Fourteen patients experienced thrombotic events (19 total; 4 arterial, 15 venous), yielding an incidence of 0.44 per 100 PY (95% CI, 0.24–0.74). No events occurred in never-treated patients; events occurred only in Groups 2–5 (*p* < 0.001). In multivariable analysis, atrial fibrillation (OR 6.01), splenectomy (OR 6.97), and higher ordinal treatment-intensity group (OR 2.16 per step) were independently associated with thrombosis (all *p* ≤ 0.027).

**Conclusion:**

Thrombosis was uncommon overall but clustered in high-risk contexts—atrial fibrillation, post-splenectomy status, and higher treatment-intensity patterns, particularly TPO-RA–based regimens. The association between treatment intensity and thrombosis most likely reflects a higher-risk clinical context—including refractory disease, greater inflammatory burden, and more intensive clinical surveillance—rather than a direct causal effect of therapy. These findings support individualized risk assessment, integrating comorbidity profiles and treatment trajectories during periods of therapy escalation and platelet instability.

**Supplementary Information:**

The online version contains supplementary material available at https://doi.org/10.1007/s00277-026-07084-z.

## Introduction

Immune thrombocytopenia (ITP) is a rare autoimmune disorder characterized by immune-mediated platelet destruction and impaired platelet production, leading to an increased risk of bleeding [1, 2]. Paradoxically, ITP patients may also exhibit an elevated risk of thrombotic events, including venous thromboembolism (VTE) and arterial thromboembolism (ATE) [3–6]. Patients with ITP exhibit a higher incidence of both arterial thromboembolism (ATE; 1.0–2.8 per 100 patient-years) and venous thromboembolism (VTE; 0.4–0.7 per 100 patient-years) compared to non-ITP populations (ATE: 0.7–1.8; VTE: 0.1–0.4), with adjusted relative risks of 1.5 (95% CI 1.3–1.8) for ATE and 1.9 (95% CI 1.4–2.7) for VTE [7].

Large population-based studies have identified advanced age, male sex, prior thrombosis, obesity, underlying thrombotic conditions, and cardiovascular comorbidities as key risk factors for thrombosis in patients with ITP [3–6, 8, 9]. Several studies have also evaluated therapy-related contributors to thrombosis in ITP, including splenectomy and exposure to specific ITP treatments [10–13]. In an Australian retrospective cohort, Goncalves et al. reported an increased thrombosis risk and identified key correlates, including advanced age, secondary ITP, multiple treatments, and TPO-RA exposure [13]. Nevertheless, important gaps remain—particularly in Asian populations and in analyses that explicitly characterize real-world treatment patterns and intensity, such as differentiating TPO-RA monotherapy from combination therapy.

Thrombotic events in ITP patients pose significant clinical challenges, as they contribute to increased morbidity and mortality [14]. Moreover, the use of antithrombotic therapies to manage thrombosis may exacerbate bleeding risk, complicating treatment decisions [15]. Understanding the incidence and risk factors for thrombosis in ITP is critical for improving patient outcomes and guiding clinical management. Given the potential influence of regional differences in patient demographics, treatment practices, and healthcare systems, investigating these risk factors in a Korean cohort is essential. This study aimed to estimate the incidence of thrombotic events in Korean adults with primary ITP and to evaluate associations with treatment intensity and patterns (including TPO-RA monotherapy vs. combination therapy) as well as key clinical risk factors (e.g., atrial fibrillation and splenectomy).

## Materials and methods

### Study design and population

This retrospective cohort study includes adult patients (aged ≥ 19 years) diagnosed with primary ITP at Seoul National University Bundang Hospital (SNUBH) between April 1, 2003, and December 31, 2023. Patients were identified through electronic medical records (EMR) based on a confirmed diagnosis of primary ITP and the availability of detailed diagnostic and follow-up data. Primary ITP was defined as thrombocytopenia with a platelet count < 100 × 10³/µL, for which no alternative cause was identified after standard clinical evaluation. The diagnosis was confirmed by a hematologist-documented diagnosis in the EMR and review of baseline clinical data. Based on clinical history and baseline assessment, we also verified that alternative causes of thrombocytopenia were unlikely, including inherited/congenital thrombocytopenia (e.g., lifelong thrombocytopenia or a suggestive family history), splenomegaly/hypersplenism, active infections, liver disease, drug-induced thrombocytopenia, pregnancy-related thrombocytopenia, hematologic malignancy or other bone marrow disorders, and other secondary causes. Exclusion criteria included age < 19 years, secondary ITP, and insufficient diagnostic or follow-up data in the EMR. Insufficient data were defined as either (i) documentation inadequate to adjudicate primary ITP (i.e., no hematologist-confirmed diagnosis and/or inability to reasonably exclude alternative causes of thrombocytopenia) or (ii) follow-up information inadequate to ascertain thrombotic outcomes and person-time at risk (e.g., no subsequent encounters after the diagnostic evaluation or missing dates for events or censoring). A total of *n* = 1,038 patients were included.

This study was approved by the Institutional Review Board of Seoul National University Bundang Hospital (IRB No. B-2504-966-106). The need for patient consent was waived by the IRB due to the retrospective nature of the study.

### Data collection and covariates

Demographic information, comorbidities, laboratory data, treatment history, and clinical outcomes were collected. Candidate risk factors were selected based on clinical relevance and prior literature. These included age, sex, BMI, smoking status, comorbidities, splenectomy, and treatment group. Cardiovascular risk (CVD risk) was assessed using a prespecified composite variable, defined as the presence of ≥ 3 of the following baseline conditions: hypertension, diabetes mellitus, dyslipidemia, and prior cardiovascular disease (angina pectoris, myocardial infarction, or stroke).

Due to the heterogeneous nature of ITP treatments, patients were categorized into five groups based on their treatment regimens for analysis. Patients in Group 1 (never treated) were those who did not receive any ITP-directed therapy (glucocorticoids, IVIg, immunosuppressants, TPO-RAs, or splenectomy) during follow-up. Group 2 comprises patients who received only first-line therapies, defined as glucocorticoids alone, intravenous immunoglobulin (IVIg) alone, or a combination of glucocorticoids and IVIg. Groups 3, 4, and 5 consist of patients who received two or more prior ITP therapies. Specifically, Group 3 includes patients treated with immunosuppressive agents (ISA), including danazol, azathioprine, cyclosporin A, dapsone, mycophenolate mofetil, cyclophosphamide, vinblastine, and vincristine, either as monotherapy or in combination with glucocorticoids and/or IVIg, without receiving TPO-RA therapy. Group 4 comprised patients treated with TPO-RA monotherapy (eltrombopag or romiplostim) during follow-up, with no concomitant ITP-directed therapy during the exposure period. Group 5 comprised patients treated with a TPO-RA in combination with other ITP therapies (glucocorticoids and/or IVIg and/or ISA) during follow-up, regardless of prior treatments. Rituximab was not used for primary ITP in this cohort because it was not reimbursed for this indication in Korea during the study period and was not used off-label at our institution; therefore, no rituximab exposure was captured.

### Outcome

The primary outcome was the occurrence of thrombotic events during the observation period. Thrombotic events were identified through diagnostic codes and confirmed by reviewing imaging studies, clinical notes, and laboratory findings. Thrombotic events were classified as either ATE or VTE. ATE included ischemic stroke, transient ischemic attack, acute myocardial infarction, peripheral arterial occlusion, and systemic arterial thromboembolism (e.g., splenic infarction, mesenteric infarction). VTE encompassed deep vein thrombosis of the lower extremities, pulmonary embolism, hepatic vein thrombosis, portal vein thrombosis, mesenteric vein thrombosis, renal vein thrombosis, ovarian vein thrombosis, cerebral vein thrombosis, and retinal vein thrombosis. Follow-up was defined as the time from the date of ITP diagnosis (the first hematologist-confirmed ITP encounter) to the first thrombotic event, death, the last recorded encounter (loss to follow-up), or the study end date (June 30, 2025), whichever occurred first.

### Statistical analysis

Baseline characteristics were summarized according to thrombosis status. Continuous variables were presented as medians with interquartile ranges (IQRs) and compared using the Wilcoxon rank-sum test. Categorical variables were summarized as counts and percentages and compared using the chi-square test or Fisher’s exact test, as appropriate. The incidence rate of thrombosis was calculated as the number of thrombotic events divided by total person-years (PY) at risk and expressed per 100 person-years, with exact Poisson 95% confidence intervals (CIs). Person-time at risk was defined from the date of first outpatient visit to the date of thrombosis, death, or last outpatient visit, whichever occurred first. Candidate variables for regression were selected based on baseline comparisons between patients with and without thrombosis (variables with *p* < 0.10). CVD risk was treated as a binary variable (yes/no) and used for subgroup incidence estimates and time-to-event curve stratification. To avoid collinearity and double counting, we did not include the individual components of the composite CVD risk variable (e.g., hypertension) in the same multivariable model; accordingly, hypertension was excluded because it is a component of CVD risk. To identify factors associated with thrombosis, we performed Firth’s penalized logistic regression. Although the data have an inherent time-to-event structure, this method was prioritized over Cox proportional hazards or Fine–Gray regression for two principal reasons: (1) the number of first thrombotic events was critically limited (*n* = 14), yielding fewer than 5 events per covariate and precluding reliable estimation of time-varying hazard functions; and (2) Firth’s method is specifically designed for rare-event settings to reduce small-sample bias and the monotone likelihood problem. Fine–Gray competing risk analysis and Kaplan–Meier estimation were retained for cumulative incidence estimation; however, multivariable time-to-event regression was not pursued as a primary inferential approach given these event-count constraints, which are acknowledged as a limitation of this study. Candidate variables with *p* < 0.10 in univariate comparisons were considered for multivariable modeling. The therapy group variable was modeled as an ordinal categorical variable reflecting treatment intensity. In regression models, Group 1 (no treatment) served as the reference category, and the reported odds ratios represent the relative increase in thrombosis risk per stepwise increment in treatment intensity from Groups 2 to 5. Thus, “therapy group” in both univariate and multivariable analyses denote all treated patients stratified by therapy intensity, rather than only advanced therapy groups. Results are reported as odds ratios (ORs) with 95% CIs.

Cumulative incidence of thrombosis was first estimated using the Kaplan–Meier (KM) method, with differences between groups assessed by the log-rank test. Cumulative incidence curves were reported as 1 − S^(t)1-\hat S(t)1 − S^(t). Because death precludes the occurrence of thrombosis, we additionally performed a competing risk analysis treating death as a competing event. The cumulative incidence function (CIF) was estimated using the method of Fine and Gray, and group differences were assessed with Gray’s test. All analyses were conducted using R (version 4.5.1) (R Foundation for Statistical Computing, Vienna, Austria). A two-sided p-value < 0.05 was considered statistically significant.

## Results

### Baseline characteristics and thrombosis events in adults with primary ITP

A total of 1038 adult patients with primary ITP were included in the study, with a median age of 57 years (IQR 40–70) and 42.6% being male (Table 1). Approximately 25.4% of patients were aged ≥ 70 years, and 35.6% had obesity (BMI ≥ 25 kg/m²). Common comorbidities included hypertension (33.2%), diabetes (18.6%), dyslipidemia (14.5%), and cardiovascular disease (6.8%). Over half of the patients (54.2%) received no ITP treatment (Group 1), while 19.5% received only first-line therapies (Group 2), 11.8% received ISA (Group 3), 9.0% received TPO-RA monotherapy (Group 4), and 5.5% received TPO-RA combination therapy (Group 5). Throughout this report, treatment group (Groups 1–5) denotes an ever-treated classification based on therapies received at any time during follow-up and is distinct from active ITP therapy status at the time of a thrombotic event; this distinction is detailed later in the event-level analysis. Splenectomy was performed in 5.5% of patients. Supplementary Table 1 compares baseline characteristics between the never-treated reference group (Group 1) and ever-treated patients (Groups 2–5), showing broadly similar cardiovascular comorbidity profiles but significantly lower baseline platelet counts in ever-treated patients (median, 24 vs. 78 × 10³/µL; *p* < 0.001), along with longer follow-up (median, 2.3 vs. 1.0 years) and differences in smoking status and cancer prevalence.

**Table 1:** Baseline characteristics by thrombosis events

		Thrombosis events		*p*
Variable	Overall (*n* = 1038)	No (*n* = 1,024)	Yes (*n* = 14)	
Age, years	57 (40–70)	57 (40–70)	57 (38–72)	0.955
$\:\:\:\ge\:\:$70, n (%)	264 (25.4)	260 (25.4)	4 (28.6)	0.761
< 70, n (%)	774 (74.6)	764 (74.6)	10 (71.4)	
Male, n (%)	442 (42.6)	434 (42.4)	8 (57.1)	0.402
BMI,$\:\mathrm{k}\mathrm{g}/\mathrm{m}^2$*	23.6 (21.4–26.3)	23.6 (21.4–26.3)	22.4 (21.2–23.3)	0.175
Obesity$\:(\mathrm{B}\mathrm{M}\mathrm{I}\:\ge\:\:25\:\mathrm{k}\mathrm{g}/\mathrm{m}^2)$, n (%)	323 (35.6)	321 (35.9)	2 (14.3%)	0.156
Smoking*				0.388
Never, n (%)	473 (75.1)	465 (75.4)	8(61.5)	
Ex-smoker, n (%)	91 (14.4)	88 (14.3)	3 (23.1)	
Current, n (%)	66 (10.5)	64 (10.4)	2 (15.4)	
Comorbidities				
Hypertension, n (%)	345 (33.2)	336 (32.8)	9 (64.3)	0.020
Diabetes, n (%)	193 (18.6)	190 (18.6)	3 (21.4)	0.732
Dyslipidemia, n (%)	150 (14.5)	147 (14.4)	3 (21.4)	0.440
Prior cardiovascular disease**, n (%)	71 (6.5)	71 (6.9)	0	0.616
Prior stroke, n (%)	40 (3.9)	39 (3.8)	1 (7.1)	0.425
Atrial fibrillation, n (%)	35 (3.4)	32 (3.1)	3 (21.4)	0.010
Cancer, n (%)	55 (5.3)	54 (5.3)	1 (7.1)	0.536
Composite CVD risk, n (%)	77 (7.4)	74 (7.2)	3 (21.4)	0.079
Treatment group**†**				< 0.001
Group 1 (Never treated), n (%)	563 (54.2)	563 (55.0)	0	
Group 2 (First-line treatment only), n (%)	202 (19.5)	199 (19.4)	3 (21.4)	
Group 3 (ISA), n (%)	123 (11.8)	120 (11.7)	3 (21.4)	
Group 4 (TPO-RA monotherapy), n (%)	93 (9.0)	90 (8.8)	3 (21.4)	
Group 5 (TPO-RA combination), n (%)	57 (5.5)	52 (5.1)	5 (35.7)	
Splenectomy, n (%)	57 (5.5)	52 (5.1)	5 (35.7)	0.001

*BMI and smoking data were available for 907 and 630 patients, respectively. **cardiovascular disease includes angina pectoris and myocardial infarction. Cardiovascular risk (CVD risk) was assessed using a prespecified composite variable, defined as the presence of ≥ 3 of the following baseline conditions: hypertension, diabetes mellitus, dyslipidemia, and prior cardiovascular disease (angina pectoris, myocardial infarction, or stroke). Patients in Group 1 (never treated) were those who did not receive any ITP-directed therapy (glucocorticoids, IVIg, immunosuppressants, TPO-RAs, or splenectomy) throughout follow-up. Group 2 comprised patients who received only first-line therapies, defined as glucocorticoids alone, IVIg alone, or a combination of glucocorticoids and IVIg. Groups 3, 4, and 5 consisted of patients who received two or more prior ITP therapies. Specifically, Group 3 included patients treated with immunosuppressive agents (danazol, azathioprine, cyclosporin A, dapsone, mycophenolate mofetil, cyclophosphamide, vinblastine, or vincristine), either as monotherapy or in combination with glucocorticoids and/or IVIg, without any TPO-RA exposure. Group 4 comprised patients treated with TPO-RA monotherapy (eltrombopag or romiplostim) during follow-up, with no concomitant ITP-directed therapy during the exposure period. Group 5 comprised patients treated with a TPO-RA in combination with other ITP therapies (glucocorticoids and/or IVIg and/or ISA) during follow-up, regardless of prior treatments

†Treatment group reflects ever-received therapy classification during follow-up; not necessarily treatment at event

Over a median follow-up of 1.4 years (IQR, 0.3–4.5), 14 patients experienced thrombotic events. Among patients who experienced thrombosis, the prevalence of hypertension was significantly higher than among those without events (64.3% vs. 32.8%; *p* = 0.020). Similarly, atrial fibrillation was more common in the thrombosis group (21.4% vs. 3.1%; *p* = 0.010). No thrombotic events were observed among patients who were never treated for ITP (Group 1). Thrombotic events occurred only among patients in Groups 2–5 (ever-treated groups), with significant differences across treatment groups (*p* < 0.001). The distribution of treatment groups differed markedly between patients with and without thrombosis. Patients with thrombosis were disproportionately represented in the groups receiving multiple or more intensive therapies, particularly TPO-RA combination therapy (i.e., TPO-RA plus glucocorticoids/IVIg and/or ISA). Additionally, splenectomy was substantially more frequent among those with thrombosis events (35.7% vs. 5.1%; *p* = 0.001).

### Thrombosis incidence and risk factors in adults with primary ITP

The incidence rates of thrombosis in 1,038 adult patients with primary ITP were evaluated over 3,185 PY, with an overall incidence rate of 0.44 per 100 PY (95% CI 0.24–0.74) (Table 2). Among specific subgroups, patients with atrial fibrillation exhibited a notably higher incidence rate of 3.58 per 100 PY (95% CI, 0.74–10.50), followed by those who underwent splenectomy at 1.40 per 100 PY (95% CI, 0.46–3.28) and those with CVD risk at 1.19 per 100 PY (95% CI, 0.25–3.47). Across treatment groups, the highest incidence was observed in Group 5 (TPO-RA combination therapy) at 1.84 per 100 PY (95% CI 0.60–4.30). No thrombosis events were reported in untreated patients (Table 2).

**Table 2:** Incidence Rates of Thrombosis

	No.	Events	PY	IR/100PY	95% CI
All patients	1,038	14	3,185	0.44	0.24–0.74
Atrial fibrillation	35	3	83.9	3.58	0.74–10.50
CVD risk (≥ 3 comorbidities)	77	3	253	1.19	0.25–3.47
Splenectomy	57	5	356	1.40	0.46–3.28
Group 2 (First-line treatment only)	202	3	508	0.59	0.12–1.73
Group 3 (ISA)	123	3	708	0.42	0.09–1.24
Group 4 (TPO-RA monotherapy)	93	3	419	0.72	0.15–2.09
Group 5 (TPO-RA combination)	57	5	271	1.84	0.60–4.30

*PY* person-years, *IR* incidence rate, *CVD* cardiovascular disease, *ISA* immunosuppressive agents, *TPO-RA*  thrombopoietin receptor agonist

CVD risk, defined as the presence of ≥ 3 of the following: hypertension, diabetes mellitus, dyslipidemia, and prior cardiovascular disease (stroke or cardiovascular disease history including angina pectoris and myocardial infarction). Group 1 includes patients who did not receive any treatment for ITP. Group 2 comprises patients who received only first-line therapies, defined as glucocorticoids alone, intravenous immunoglobulin (IVIg) alone, or a combination of glucocorticoids and IVIg. Groups 3, 4, and 5 consist of patients who received two or more prior ITP therapies. Specifically, Group 3 includes patients treated with immunosuppressive agents (ISA) either as monotherapy or in combination with glucocorticoids and/or IVIg, without receiving TPO-RA therapy. Group 4 comprises patients treated with TPO-RA monotherapy, regardless of prior treatments. Group 5 includes patients who received TPO-RA in combination with other therapies, irrespective of any previous treatments

Firth logistic regression analysis was conducted to identify risk factors for thrombosis in 1,038 Korean adult patients with primary ITP (Table 3). Univariate analysis revealed significant associations with atrial fibrillation (OR 9.29, 95% CI 2.28–29.74, *p* = 0.004), splenectomy (OR 10.72, 95% CI 3.38–31.03, *p* < 0.001), and therapy group (OR 2.43, 95% CI 1.69–3.68, *p* < 0.001; reference: no treatment), while CVD risk, defined as ≥ 3 comorbidities (hypertension, diabetes mellitus, dyslipidemia, or prior cardiovascular disease), showed a borderline association (OR 3.88, 95% CI 0.97–12.03, *p* = 0.054). In multivariable analysis, atrial fibrillation (OR 6.01, 95% CI 1.25–23.87; *p* = 0.027), splenectomy (OR 6.97, 95% CI 2.06–22.24; *p* = 0.003), and therapy group (OR 2.16, 95% CI 1.46–3.33; *p* < 0.001; reference: no treatment) were independently associated with thrombosis. A stepwise increase in event rates was observed across ordinal treatment-intensity categories. Importantly, this stepwise pattern should not be interpreted as evidence of a causal treatment effect; rather, it most likely reflects confounding by disease severity, whereby patients requiring escalated or combination therapy represent a higher-risk clinical subgroup with greater comorbidity burden, more refractory disease, and more frequent clinical contact—any of which may contribute to increased thrombosis risk and detection. CVD risk was not statistically significant in the multivariable model (OR 3.15, 95% CI 0.66–11.79, *p* = 0.137). These findings highlight atrial fibrillation, splenectomy, and specific ITP treatments, particularly TPO-RA-based therapies, as key risk factors for thrombosis in this cohort.

**Table 3:** Univariate and Multivariate Firth Logistic Regression Analysis of Thrombosis Risk Factors

	Univariate		Multivariate	
Variable	OR (95% CI)	*p*	OR (95% CI)	*p*
Atrial fibrillation	9.29 (2.28–29.74)	0.004	6.01 (1.25–23.87)	0.027
CVD risk (≥ 3 comorbidities)	3.88 (0.97–12.03)	0.054	3.15 (0.66–11.79)	0.137
Splenectomy	10.72 (3.38–31.03)	< 0.001	6.97 (2.06–22.24)	0.003
Therapy group	2.43 (1.69–3.68)	< 0.001	2.16 (1.46–3.33)	< 0.001

CVD risk, defined as the presence of ≥ 3 of the following: hypertension, diabetes mellitus, dyslipidemia, and prior cardiovascular disease (stroke or cardiovascular disease history including angina pectoris and myocardial infarction)

**Fig. 1:**
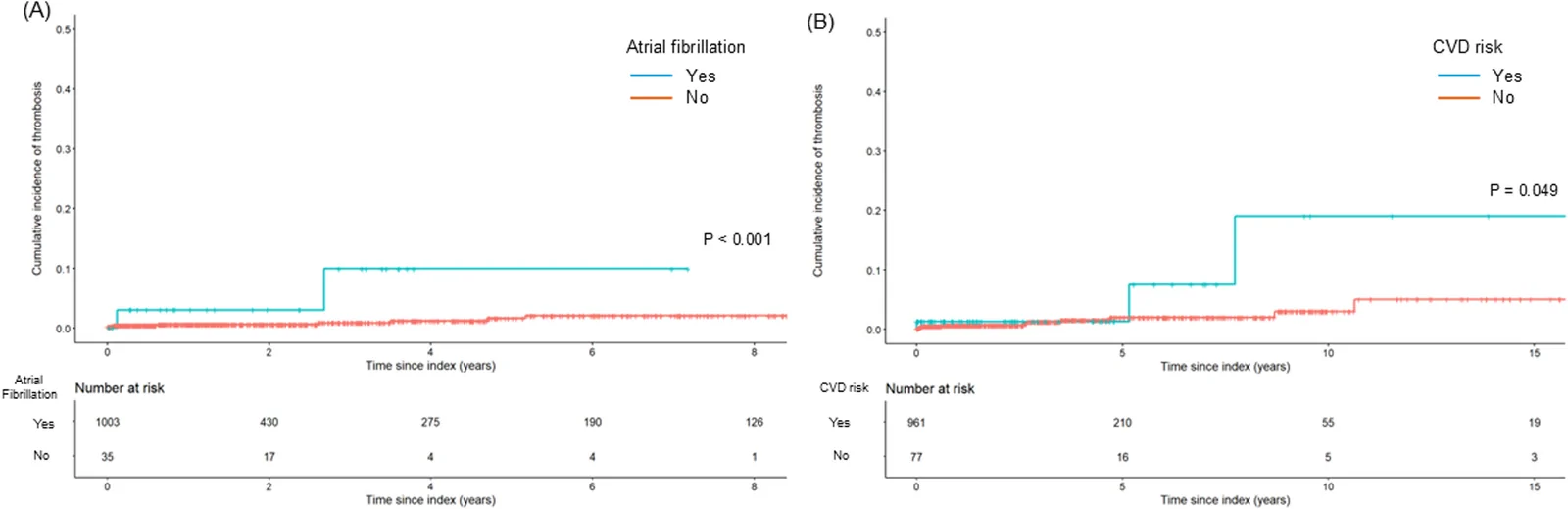
Kaplan–Meier curves for cumulative incidence of thrombosis by baseline comorbidity. (**A**) Atrial fibrillation. (**B**) CVD risk (defined as ≥ 3 of hypertension, diabetes mellitus, dyslipidemia, and prior cardiovascular disease)

**Fig. 2:**
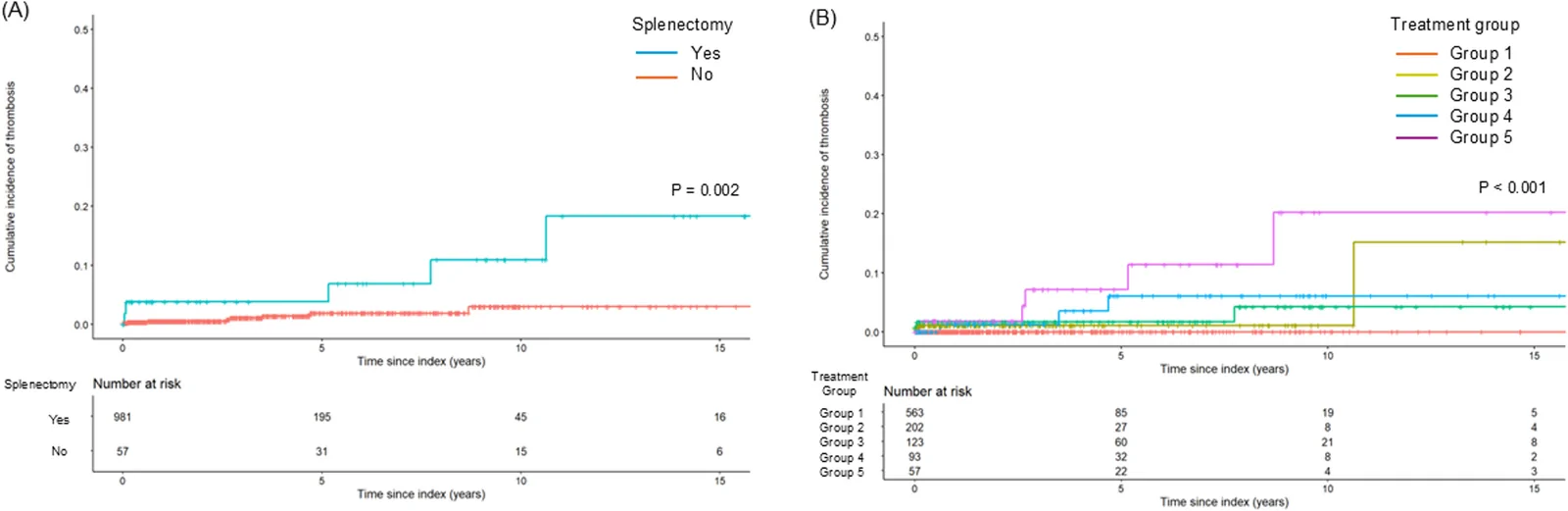
Kaplan–Meier Curves for Cumulative Incidence of Thrombosis by Treatment. (**A**) Splenectomy (**B**) Treatment group

Kaplan-Meier analysis illustrated the cumulative incidence of thrombosis in adult patients with primary ITP, stratified by key risk factors. Patients with atrial fibrillation exhibited a significantly higher cumulative incidence compared to those without (*p* < 0.001) (Fig. 1A). Similarly, patients with CVD risk (defined as the presence of ≥ 3 of the following: hypertension, diabetes mellitus, dyslipidemia, and prior cardiovascular disease) had a higher cumulative incidence than those without CVD risk (*p* = 0.049), although the difference was less pronounced (Fig. 1B). Splenectomy was associated with a markedly increased risk, as evidenced by the divergent curves (*p* = 0.002), with splenectomized patients demonstrating a sharper rise in incidence over time (Fig. 2A). Across treatment groups, cumulative incidence increased stepwise from Group 1 (never treated) to Group 5 (TPO-RA combination therapy), with significant overall differences (*p* < 0.001) (Fig. 2B).

### Thrombosis outcomes

Detailed clinical data and treatment outcomes for all 14 patients with thrombotic events are presented in Table 4. Among the 14 patients, 19 thrombotic events were recorded in total: 4 ATE (3 strokes, 1 splenic infarction) and 15 VTE (6 pulmonary embolism, 5 DVT, 3 DVT combined with pulmonary embolism, 1 portal vein thrombosis). At the time of thrombosis, 11 of 14 patients were receiving active ITP-directed therapy (Group 5, *n* = 5; Group 4, *n* = 3; Group 3, *n* = 2; Group 2, *n* = 1), while three patients (Cases 1, 2, and 4) experienced events outside of active ITP treatment—due to subtherapeutic anticoagulation in the setting of a mechanical valve (Case 2), antiplatelet therapy for atrial fibrillation (Case 1), and post-splenectomy status following treatment failure (Case 4).

Several recurring clinical patterns were apparent across cases. First, events in actively treated patients predominantly occurred during periods of platelet instability, often in the context of refractory ITP requiring multimodal therapy. Second, anticoagulation was frequently interrupted, dose-reduced, or of abbreviated duration due to concurrent severe thrombocytopenia or clinically significant bleeding. Third, patients with recurrent thrombosis (Cases 6, 8, 9, and 11) experienced re-events despite prior anticoagulation exposure, underscoring the difficulty of sustaining therapeutic anticoagulation in the context of thrombocytopenia. Fourth, two patients (Cases 7 and 9) were receiving oral contraceptives for heavy menstrual bleeding (HMB) at the time of thrombosis. All but one patient received anticoagulation for their thrombotic events; the exception was a patient with splenic infarction and severe thrombocytopenia (platelet count 26 × 10³/µL) in whom anticoagulation was withheld. Full clinical details are provided in Table 4.

**Table 4:** Clinical characteristics and antithrombotic management at the time of thrombosis (event-level) in patients with primary ITP

Pt No.	Age (y)	Sex	AF	Composite CVD risk*	Splenectomy	Ever-treated group (1–5)†	Thrombosis event No. (per patient)	Event type	Event details	Active ITP therapy at event‡ (regimen)	PLT at Event (×10³/µL)	Treatment for Thrombosis
1	75	M	Y	Y	Y	3	1	ATE	Stroke	N	193	TFCA, apixaban
2	38	F	N	N	Y	2	1	ATE	Stroke	N	67	Warfarin
3	80	F	N	N	N	4	1	VTE	PE	Y (eltrombopag)	34	Apixaban for 3 years, then switched to clopidogrel
4	22	M	N	N	Y	2	1	VTE	PVT	N	519	Warfarin for 4 months
5	82	F	N	N	Y	3	1	VTE	DVT	Y (prednisolone + azathioprine)	264	Thrombolysis, warfarin for 5 months
6	61	M	Y	N	N	5	2	VTE	1st (DVT), 2nd (PE)	Y (1st prednisolone + danazol + eltrombopag) (2nd prednisolone + romiplostim + IVIg)	1st (75), 2nd (209)	1st (thrombectomy, warfarin for 5 months, then switched to aspirin), 2nd (edoxaban for 6 months)
7	47	F	N	N	N	3	1	VTE	DVT	Y (azathioprine)	168	Rivaroxaban for 3 months
8	34	M	N	N	N	5	2	1st (ATE), 2nd (VTE)	1st (stroke), 2nd (DVT/PE)	Y (1st prednisolone + romiplostim + IVIg) (2nd prednisolone + eltrombopag)	1st (344), 2nd (59)	1st (aspirin for 1 month), 2nd (rivaroxaban, then switched to warfarin)
9	41	F	N	N	N	4	2	1st (VTE), 2nd (VTE)	1st (DVT), 2nd (DVT/PE)	Y (1st eltrombopag) (2nd eltrombopag)	1st (22), 2nd (54)	1st (apixaban for 2months), 2nd (apixaban)
10	22	M	N	N	N	4	1	VTE	DVT	Y (eltrombopag)	22	Apixaban for 9 months
11	57	F	N	Y	Y	5	3	1st (VTE), 2nd (VTE), 3rd (VTE)	1st (PE), 2nd (PE), 3rd (PE)	Y (1st ~3rd prednisolone + romiplostim + cyclosporin + IVIg)	1st (917), 2nd (410), 3rd (286)	1st (rivaroxaban for 15 days), 2nd (rivaroxaban for 1 month), 3rd (enoxaparin for 3 months)
12	43	M	N	Y	N	2	1	ATE	Splenic infarction	Y (prednisolone + IVIg)	N/A	Anticoagulation held due to thrombocytopenia
13	72	M	N	N	N	5	1	VTE	DVT/PE	Y (prednisolone + danazol + romiplostim)	166	Rivaroxaban
14	55	M	Y	N	N	5	1	VTE	PE	Y (prednisolone + danazol + eltrombopag)	89	VA-ECMO insertion, tPA thrombolysis, rivaroxaban

ITP, immune thrombocytopenic purpura; PLT, platelet count; AF, atrial fibrillation; CVD, cardiovascular disease; ATE, arterial thromboembolism; VTE, venous thromboembolism; PE, pulmonary embolism; DVT, deep vein thrombosis; PVT, portal vein thrombosis; TFCA, transfemoral cerebral angiography; VA-ECMO, veno-arterial extracorporeal membrane oxygenation; tPA, tissue plasminogen activator; DOAC, direct oral anticoagulant; N/A, not available

* Composite CVD risk was defined as the presence of ≥ 3 of the following: hypertension, diabetes mellitus, dyslipidemia, and prior cardiovascular disease (stroke and/or ischemic heart disease including angina pectoris or myocardial infarction)

† Ever-treated group (Groups 1–5) indicates treatment classification based on therapies received at any time during follow-up: Group 1, never treated; Group 2, first-line therapy only (glucocorticoids and/or IVIg); Group 3, immunosuppressive agents without TPO-RA; Group 4, TPO-RA monotherapy; Group 5, TPO-RA combination therapy

‡ Active ITP therapy at event indicates whether the patient was receiving ITP-directed therapy at the time of thrombosis. This variable is distinct from the ever-treated group classification

## Discussion

This 20-year retrospective cohort study of 1,038 Korean adults with primary ITP provides real-world estimates of thrombotic event incidence and identifies clinical and treatment-related factors associated with thrombosis. While thrombosis in ITP has been reported across multiple populations, data from Asian cohorts remain limited, and treatment availability and patterns may differ by region and over time. Our results complement the existing literature by quantifying thrombotic incidence in a Korean tertiary-care setting and by examining treatment patterns—including TPO-RA monotherapy versus combination therapy—in relation to thrombotic risk.

The overall thrombosis incidence of 0.44 per 100 PY in our Korean primary ITP cohort was somewhat lower than those reported in prior non-Asian studies, such as 1.5–1.6 per 100 PY for ATE and 0.7–0.9 per 100 PY for VTE [16, 17]. Our cohort exhibited a predominance of VTE events (79% of cases), consistent with meta-analyses indicating a higher VTE burden in ITP [7]. When compared to the general Korean population, the overall thrombosis incidence in our primary ITP cohort appeared substantially elevated, with VTE rates in the general population estimated at 0.054 per 100 PY in 2018 [18], and ATE incidence (primarily acute myocardial infarction) around 0.04 per 100 PY in 2016 [19]. This difference should be interpreted cautiously, as incidence estimates in ITP are sensitive to cohort composition (e.g., disease severity distribution, referral patterns), comorbidity burden, surveillance intensity, and study definitions. Nevertheless, consistent with prior reports, venous thromboembolism predominated over arterial events in our cohort. Importantly, our findings should not be interpreted as evidence that Korean patients with ITP are protected from thrombosis; rather, they indicate that in this real-world cohort, thrombotic events were uncommon overall but concentrated in clinically recognizable high-risk subgroups.

A key finding was the association between thrombosis and both traditional risk factors and ITP-related management. Atrial fibrillation was strongly associated with thrombosis (OR 6.01; incidence 3.58 per 100 PY), consistent with its established prothrombotic role and the clinical complexity of anticoagulation in patients with fluctuating platelet counts [20]. In contrast, the composite CVD risk (≥ 3 comorbidities) showed only borderline univariate significance (OR 3.88, *p* = 0.054) and was not statistically significant in the multivariable model, suggesting that individual factors such as atrial fibrillation may contribute more to risk than cumulative comorbidity burden in this population. Splenectomy was also associated with an increased risk of thrombosis (OR 6.97; incidence 1.40 per 100 PY), likely through multiple mechanisms, including post-splenectomy thrombocytosis, altered splanchnic/portal hemodynamics, and a procoagulant shift characterized by increased circulating cell-derived microparticles in splenectomized patients with ITP [21–23]. These results reinforce the need to consider baseline thrombotic risk profiles when making ITP treatment decisions and when planning post-splenectomy monitoring.

With respect to ITP therapy, we observed that thrombotic events occurred only among patients classified as ever treated (Groups 2–5), with a stepwise increase in incidence across ordinal treatment-intensity groups and the highest incidence in the TPO-RA combination therapy group (Group 5; 1.84 per 100 PY). This pattern should be interpreted as an association rather than a causal effect of therapy. In routine care, escalation to multiple or advanced therapies often reflects refractory disease, greater inflammatory burden, greater exposure to transient risk factors (e.g., hospitalization, procedures), and more frequent clinical contacts, all of which may increase the likelihood of thrombosis detection and may confound the relationship between therapy and thrombosis. Consistent with confounding by disease severity, ever-treated patients had substantially lower baseline platelet counts at diagnosis than never-treated patients (Supplementary Table 1), suggesting a more severe initial phenotype and a higher likelihood of subsequent treatment escalation. This baseline imbalance should be considered when interpreting the observed association between treatment intensity and thrombosis. Treatment-group associations should be interpreted in the context of Korean reimbursement policies during the study period, in which TPO-RAs were generally reserved for patients refractory to glucocorticoids and/or IVIg and coverage required either prior splenectomy or a documented contraindication to splenectomy. This policy-driven sequencing may have enriched the TPO-RA groups for more refractory disease and confounded the observed association between treatment intensity and thrombosis. Nonetheless, our stratification by TPO-RA monotherapy versus combination therapy suggests that patients requiring combination regimens represent a particularly vulnerable subgroup, warranting careful risk assessment and closer surveillance during periods of platelet instability and active treatment intensification. The EXTEND study reported a thrombosis incidence of 2.69 per 100 PY during TPO-RA therapy [24]. Although these findings await confirmation from rigorous randomized trials, annualized thrombosis rates in adults seem 2- to 3-fold higher with TPO-RA treatment compared to untreated ITP patients [25]. The underlying mechanisms driving this heightened thrombotic risk with TPO-RA remain incompletely understood [26]. Broadly, TPO-RA therapy heightens thrombotic risk in ITP patients by augmenting platelet activation and thrombin generation, elevating plasminogen activator inhibitor-1 levels, and inducing platelet apoptosis, thereby establishing a procoagulant milieu that promotes thrombus formation [27, 28]. Importantly, treatment group assignment (Groups 1–5) represents an ever-treated classification during follow-up and is distinct from active ITP therapy status at the time of thrombosis: no events occurred among patients who were never treated for ITP (Group 1), whereas several events occurred when patients were not receiving active ITP therapy at the time of thrombosis, reflecting prior treatment exposure and/or post-splenectomy status rather than belonging to the never-treated group. This distinction is clinically important because thrombotic risk in ITP may persist beyond discrete treatment windows and may be influenced by both prior interventions and evolving comorbidities over long follow-up.

Our case descriptions highlight the practical dilemma of balancing thrombotic recurrence risk against bleeding risk in ITP. In several patients—particularly those with refractory ITP requiring multimodal therapy—anticoagulation intensity and duration were constrained by severe thrombocytopenia and/or clinically significant bleeding. Therefore, the antithrombotic strategies observed in this study should not be construed as prescriptive; they reflect real-world decisions made under competing risks. Because a history of thrombosis is a strong predictor of recurrence, anticoagulation interruption or early discontinuation may plausibly increase recurrence risk, particularly in patients with recurrent events or atrial fibrillation [29, 30]. We also acknowledge that interruptions or de-escalations of anticoagulation may contribute to recurrence risk and could influence observed event patterns in retrospective cohorts. Notably, in selected patients, TPO-RAs may be continued or re-introduced as platelet support to maintain a platelet count compatible with therapeutic anticoagulation, underscoring the need for individualized management [15]. Contemporary strategies such as left atrial appendage closure were not used in this cohort and were not routinely applied across the long study period; their potential role in selected high-risk patients with contraindications to long-term anticoagulation merits further evaluation.

Several limitations should be considered. First, this was a single-center study at a tertiary referral hospital, which may limit generalizability and may enrich for more complex or refractory cases. Second, although thrombotic outcomes were confirmed by review of imaging and clinical documentation, retrospective ascertainment may still be influenced by coding practices and incomplete records. Third, the small number of thrombotic events resulted in wide confidence intervals and limited power to evaluate less frequent covariates or specific therapy subtypes. Additionally, the limited number of first thrombotic events (*n* = 14) precluded the use of multivariable time-to-event regression (Cox or Fine–Gray models) as a primary analytical approach; the reported odds ratios from Firth’s penalized logistic regression should therefore be interpreted as associative estimates, and findings should be validated in larger prospective or multicenter cohorts. Fourth, baseline characteristics differed between never-treated and ever-treated patients, including substantially lower baseline platelet counts at diagnosis, longer follow-up, and a higher prevalence of cancer in the ever-treated group (Supplementary Table 1), which may introduce confounding by indication/severity and detection bias when interpreting treatment–thrombosis associations. Fifth, rituximab was not used in this cohort due to local reimbursement and prescribing constraints, which may limit direct comparisons with settings where rituximab is commonly used. Sixth, antiphospholipid antibody testing was not systematically available; this is clinically relevant because antiphospholipid syndrome (APS) can present with venous or arterial thrombosis, particularly in younger patients, and unrecognized APS may have contributed to thrombotic events in a subset of patients [31, 32]. This lack of standardized APS assessment may introduce residual confounding and lead to either underestimation or overestimation of associations between ITP-related factors (including treatment intensity) and thrombosis risk; future multicenter studies should incorporate standardized APS evaluation and centralized, protocol-driven event adjudication. Finally, antithrombotic management was heterogeneous and frequently constrained by thrombocytopenia and bleeding, which may have influenced recurrence risk and event ascertainment, thereby complicating causal interpretation of treatment–thrombosis associations; moreover, ITP treatment practices and availability evolved over the two-decade study period, introducing additional temporal heterogeneity that cannot be fully addressed in this observational design.

In conclusion, in this Korean adult primary ITP cohort, thrombotic events were uncommon overall but clustered in high-risk clinical contexts, particularly atrial fibrillation, post-splenectomy status, and higher treatment-intensity patterns including TPO-RA–based regimens. The association between treatment intensity and thrombosis most likely reflects a higher-risk clinical context—characterized by refractory disease, greater inflammatory burden, lower baseline platelet counts, and more frequent clinical contacts—rather than a direct causal effect of the therapies themselves. These findings support a pragmatic approach to care: clinicians should integrate comorbidity profiles and treatment trajectories into individualized thrombotic risk assessment, especially during periods of therapy escalation and platelet instability.

## Supplementary Information

Below is the link to the electronic supplementary material.

Supplementary Material 1

## Data Availability

The datasets generated and/or analyzed during the current study are not publicly available due to patient confidentiality and institutional data protection policies. De-identified data may be made available from the corresponding author upon reasonable request and with approval from the Institutional Review Board of the institution.
