# Supplementary material for: Thrombosis in adult primary immune thrombocytopenia in Korea: incidence and risk factors in a 20-year retrospective cohort
Source: Ann Hematol. 2026 Jun 8;105(9):381. doi: 10.1007/s00277-026-07084-z (PMC13481474; doi:10.1007/s00277-026-07084-z)
Supplement: Supplementary file 1 [file 277_2026_7084_MOESM1_ESM.docx]

**Table S1. Baseline characteristics by treatment exposure (never treated vs ever treated)**

| **Variable** | **Group 1**  **Never treated**  **(n = 563)** | **Groups 2–5**  **Ever treated**  **(n = 475)** | **p** |
| --- | --- | --- | --- |
| Age, years, (median, IQR) | 58 (43–70) | 54 (37–70) | 0.152 |
| $\mathrm{Age} \geq$70, n (%) | 143 (25.4) | 121 (25.5) | 0.999 |
| Male, n (%) | 253 (44.9) | 189 (39.8) | 0.108 |
| BMI, $kg/m^{2}$ (median, IQR)* | 23.9 (21.8–26.2) | 23.3 (21.2–26.3) | 0.074 |
| Obesity $(BMI \geq25 kg/m^{2})$, n (%) | 175 (37.6) | 148 (33.5%) | 0.217 |
| Smoking, n (%)* |  |  | 0.016 |
| Never | 212 (71.6) | 216 (78.1) |  |
| Ex-smoker | 42 (14.2) | 24 (7.2) |  |
| Current | 42 (14.2) | 49 (14.7) |  |
| Platelet count (×10³/μL), (median, IQR) | 78 (57–92) | 24 (10–52) | <.001 |
| Comorbidities |  |  |  |
| Hypertension, n (%) | 181 (32.1) | 164 (34.5) | 0.457 |
| Diabetes, n (%) | 105 (18.7) | 88 (18.5) | 0.999 |
| Dyslipidemia, n (%) | 80 (14.2) | 70 (14.7) | 0.879 |
| Prior cardiovascular disease, n (%)** | 44 (7.8) | 27 (5.7) | 0.218 |
| Prior stroke, n (%) | 24 (4.3) | 16 (3.4) | 0.559 |
| Atrial fibrillation, n (%) | 17 (3.0) | 18 (3.8) | 0.609 |
| Cancer, n (%) | 19 (3.4) | 36 (7.6) | 0.004 |
| Composite CVD risk, n (%) | 42 (7.5) | 35 (7.4) | 0.999 |
| Follow-up, years (median, IQR) | 1.0 (0.1–3.1) | 2.3 (0.6–6.4) | <.001 |

*BMI and smoking data were available for 907 and 630 patients, respectively. **cardiovascular disease includes angina pectoris and myocardial infarction. Cardiovascular risk (CVD risk) was assessed using a prespecified composite variable, defined as the presence of ≥3 of the following baseline conditions: hypertension, diabetes mellitus, dyslipidemia, and prior cardiovascular disease (history of stroke, angina pectoris, or myocardial infarction).

**Statistical tests:** Continuous variables were compared using the Wilcoxon rank-sum test, and categorical variables using the chi-square test or Fisher’s exact test, as appropriate.
